# Comparative Phytochemical Profiling and In Vitro Investigation of the Antioxidant and Antimicrobial Potential of *Arnica montana* L., *Melissa officinalis* L. and *Capsella bursa-pastoris* Medik. Extracts and Their Synergistic Combinations

**DOI:** 10.3390/molecules31101735

**Published:** 2026-05-19

**Authors:** Sorina-Georgiana Onea Mînz, Cristina Burlou-Nagy Fati, Neli Kinga Olah, Anett Jolan Karetka, Rodica Anamaria Negrean, Mariana Ganea, Olimpia Daniela Frent, Florin Banica, Annamaria Pallag

**Affiliations:** 1 Doctoral School of Biomedical Sciences, Faculty of Medicine and Pharmacy, University of Oradea, 410073 Oradea, Romania; onea.sorinageorgiana@student.uoradea.ro (S.-G.O.M.); balasko.anettjolan@student.uoradea.ro (A.J.K.); apallag@uoradea.ro (A.P.); 2 Department of Pharmacy, Faculty of Medicine and Pharmacy, University of Oradea, 410073 Oradea, Romania; mganea@uoradea.ro (M.G.); ofrent@uoradea.ro (O.D.F.); fbanica@uoradea.ro (F.B.); 3 Department of Pharmaceutical Chemistry, Faculty of Pharmacy, Vasile Goldis Western University of Arad, 310414 Arad, Romania; neliolah@yahoo.com; 4 PlantExtrakt Ltd., 407059 Cluj-Napoca, Romania; 5 Department of Preclinical Disciplines, Faculty of Medicine and Pharmacy, University of Oradea, 410073 Oradea, Romania; rodicanegrean@uoradea.ro

**Keywords:** antimicrobial effect, antioxidant activity, anthocyanin, combinations, extracts, flavonoids, LC/MS, polyphenols

## Abstract

This study investigated the phytochemical composition, antioxidant potential, and antimicrobial activity of ethanolic extracts obtained from *Arnica montana* L., *Melissa officinalis* L., and *Capsella bursa-pastoris* Medik., as well as their ternary mixtures. Liquid chromatography coupled with mass spectrometry analysis revealed the presence of several phenolic compounds, including luteolin, apigenin, acacetin, and phenolic acids, while rutin and hyperoside were previously reported as dominant constituents in *Capsella bursa-pastoris* Medik. The extracts and their mixtures exhibited significant antioxidant activity in different radical scavenging and reducing power assays, with the highest activity observed for the ACM4 mixture. Antimicrobial activity was evaluated against *Staphylococcus aureus* and *Streptococcus pneumoniae*, showing inhibitory effects with minimum inhibitory concentration values ranging from below 100 mg/L for *Melissa officinalis* L. extracts to above 250 mg/L for *Capsella bursa-pastoris* Medik. extracts. These findings suggest that the phenolic compounds identified in the studied plants contribute to their antioxidant and antibacterial properties and support the potential use of these extracts and their combinations as natural sources of bioactive compounds.

## 1. Introduction

Medicinal plants represent an important source of bioactive compounds with antioxidant and antimicrobial properties, attracting considerable interest for their potential applications in pharmaceutical, nutraceutical, and food systems. Phenolic compounds, particularly flavonoids and phenolic acids, are recognized as key contributors to the biological activity of plant extracts due to their capacity to neutralize reactive oxygen species and inhibit the growth of pathogenic microorganisms [1,2]. Among medicinal species widely used in traditional European phytotherapy, *Arnica montana* L., *Melissa officinalis* L., and *Capsella bursa-pastoris* Medik. are notable for their diverse phytochemical composition and biological effects. Recent studies have shown that *Arnica montana* L. extracts are rich in hydroxycinnamic derivatives, particularly caffeoylquinic acids, which contribute to their antioxidant and antimicrobial activity [3]. Similarly, *Melissa officinalis* L. contains high levels of phenolic compounds, especially rosmarinic acid and flavonoids, associated with strong antioxidant and antibacterial properties [4]. *Capsella bursa-pastoris* has been reported to contain glucosinolates, flavonoids, and phenolic acids, which contribute to its antioxidant, hepatoprotective, and antimicrobial activities [5,6].

Although the phytochemical composition and biological activity of these plants have been investigated individually, limited data are available regarding the potential synergistic effects resulting from their combined use. Increasing evidence suggests that mixtures of plant extracts may enhance antioxidant and antimicrobial activity through additive or synergistic interactions among different phenolic constituents [1,2]. However, limited information is available regarding the combined biological activity of *Arnica montana* L., *Melissa officinalis* L., and *Capsella bursa-pastoris* Medik. extracts, particularly in relation to their phytochemical composition and potential synergistic effects.

The aim of this study was to characterize extracts from C.B.P., M.O., and A.M. in terms of their content of bioactive compounds, polyphenols, and flavonoids, antioxidant capacity, quantitative determination of compounds and determination of antimicrobial effect. The best results for these extracts will be used to make mixtures of the extracts and determine their antioxidant capacity and antimicrobial effect. The optimal concentrations resulting from the analysis of the mixtures will be used in the next study to create a pharmaceutical formulation.

## 2. Results

### 2.1. The Content of Polyphenols, Flavonoids and Anthocyanin in the Studied Plant Extracts

The quantitative analysis of bioactive compounds revealed noticeable variations among the extracts obtained from *Capsella bursa-pastoris* Medik., *Melissa officinalis* L., and *Arnica montana* L. (Table 1).

The quantitative phytochemical evaluation revealed significant variations in the concentration of secondary metabolites among the three plant species investigated. As shown in Table 1, *Arnica montana* L. (A.M.) exhibited the highest total polyphenol content (426.12 mg GAE/g DW), followed by *Capsella bursa-pastoris* Medik. (C.B.P.) (368.17 mg GAE/g DW) and *Melissa officinalis* L. (M.O.) (352.73 mg GAE/g DW). A similar trend was observed for total flavonoid content, with A.M. displaying the highest concentration (24.53 mg QE/g DW), whereas M.O. and C.B.P. presented moderately lower values (20.08 and 18.84 mg QE/g DW, respectively).

Anthocyanin levels ranged from 1.1250 to 3.4740 mg C3G/100 g DW, with A.M. again presenting the highest content (3.4740 mg C3G/100 g DW), followed by C.B.P. (2.7013 mg C3G/100 g DW) and M.O. (1.1250 mg C3G/100 g DW). These findings indicate substantial interspecific differences in the accumulation of phenolic and flavonoid constituents.

### 2.2. Antioxidant Capacity for Capsella bursa-pastoris Medik., Arnica montana L., Melissa officinalis L. Species

Thus, we evaluated the antioxidant activity of the plant extract using the DPPH (Brand-Williams et al. (1995) [7]), ABTS, CUPRAC and FRAP methods. The results obtained are summarized in Table 2. In the case of *Melissa officinalis* L. extract, higher values were obtained compared to the literature, where the value was 46.71% [8].

The antioxidant capacity of the individual extracts was assessed using four complementary assays (DPPH, CUPRAC, ABTS, and FRAP). All species demonstrated strong antioxidant activity, although the magnitude varied between methods. In the DPPH assay, the scavenging activity ranged from 84% (C.B.P.) to 92% (A.M.). CUPRAC values ranged from 358 to 538 μmol TE/mL, with the highest activity recorded for A.M. (538 μmol TE/mL) and M.O. (516 μmol TE/mL). ABTS radical-scavenging capacity followed a similar pattern, ranging between 280 and 331 μmol TE/mL. In the FRAP assay, A.M. (632 μmol TE/100 g) and C.B.P. (617 μmol TE/100 g) exhibited the strongest ferric-reducing activity, while M.O. showed a slightly lower value (573 μmol TE/100 g).

Collectively, all extracts showed robust antioxidant activity, with notable assay-dependent distinctions.

### 2.3. LC-MS Analysis

The obtained qualitative and quantitative results are presented in Table 3.

LC-MS analysis identified multiple phenolic acids and flavonoids in both A.M. and M.O., with marked quantitative differences between the species (Table 3). A.M. displayed high concentrations of luteolin-7-O-glucoside (121.8 μg/mL), tilianin (11.707 mg/mL), and quercetin (22.807 μg/mL), along with significant amounts of caffeic and chlorogenic acid derivatives.

M.O. exhibited lower concentrations of most of these components, with the exception of acacetin (3.259 μg/mL), which was present at higher levels than in A.M. Certain compounds, such as apigenin and luteolin-7-O-rutinoside, were below the limit of quantification in both species.

The biological activities observed in the studied extracts may be associated with the presence of phenolic acids and flavonoids identified in Table 3. Compounds such as caffeic acid, chlorogenic acid and their derivatives are well known for their antioxidant properties, mainly due to their ability to donate hydrogen atoms and scavenge free radicals. In addition, flavonoids such as quercetin, luteolin-7-O-glucoside, tilianin and acacetin have been widely reported to exhibit both antioxidant and antimicrobial activities. The relatively higher concentrations of luteolin-7-O-glucoside and quercetin in the analyzed samples may contribute significantly to the observed biological effects.

The LC–MS analysis of *Capsella bursa-pastoris* Medik. extract revealed a phytochemical profile characterized predominantly by phenolic acids and moderate levels of flavonoids. Among the identified compounds, chlorogenic acid was the major phenolic constituent (4.015 ± 0.0075 μg/mL), accompanied by its isomers, namely neochlorogenic acid (1.097 ± 0.0198 μg/mL) and cryptochlorogenic acid (1.081 ± 0.1260 μg/mL), as well as caffeic acid (2.324 ± 0.0123 μg/mL). Regarding flavonoids, quercetin (12.445 ± 0.0520 μg/mL), tilianin (6.357 ± 0.0065 μg/mL), and luteolin-7-O-glucoside (7.305 ± 0.0550 μg/mL) were identified as the main constituents, while lower concentrations were recorded for luteolin-7-O-rutioside (1.047 ± 0.0071 μg/mL) and acacetin (0.310 ± 0.1032 μg/mL). Several compounds, including apigenin, hesperetin, and naringenin, were detected below the limit of quantification. Overall, the quantitative profile indicates that C.B.P. contains a diverse but moderately concentrated pool of bioactive compounds, with phenolic acids likely contributing substantially to its antioxidant capacity, while the relatively lower flavonoid content may explain its reduced antimicrobial activity compared to the other studied species.

### 2.4. Antimicrobial Activity for Capsella bursa-pastoris Medik., Arnica montana L., Melissa officinalis L. Species

The antimicrobial activity of *Melissa officinalis* L., *Arnica montana* L., and *Capsella bursa-pastoris* Medik. extracts were evaluated against *Staphylococcus aureus* ATCC 25923 and *Streptococcus pneumoniae* ATCC 49619 by disk diffusion and MIC determination, and the results are presented in Table 4.

The antimicrobial activity of *Melissa officinalis* L., *Arnica montana* L., and *Capsella bursa-pastoris* Medik. extracts were evaluated against *Staphylococcus aureus* ATCC 25923 and *Streptococcus pneumoniae* ATCC 49619 by disk diffusion. Disk diffusion results revealed a clear concentration–response relationship, with larger inhibition zones observed at lower dilution ratios. Among the three extracts, M.O exhibited the strongest activity overall. At 1:4 dilution, M.O produced the largest inhibition zones against *Staphylococcus aureus* (20 mm) and *Streptococcus pneumoniae* (18 mm), approaching the activity of standard antibiotics such as gentamicin (22 mm) and oxacillin (22 mm). AM showed moderate inhibition (12–15 mm at 1:4), while C.B.P produced the smallest zones (10–12 mm).

### 2.5. Antioxidant Capacity for Mixture of Capsella bursa-pastoris Medik., Melissa officinalis L., Arnica montana L.

All extract mixtures (ACM1–ACM7) demonstrated considerable antioxidant activity in the DPPH assay. The inhibition percentage ranged between 67.23% and 75.34%, with formulation ACM4 (1:1:2; A.M.:C.B.P.:M.O.) showing the highest activity (75.34%). ACM2 and ACM1 also exhibited elevated antioxidant capacities (72.68% and 71.48%, respectively). Increasing the volume of the extract did not significantly enhance inhibitory activity, suggesting saturation of active compounds.

The results are presented in Table 5.

### 2.6. Antimicrobial Activity for Mixture of Species

**Figure 1:**
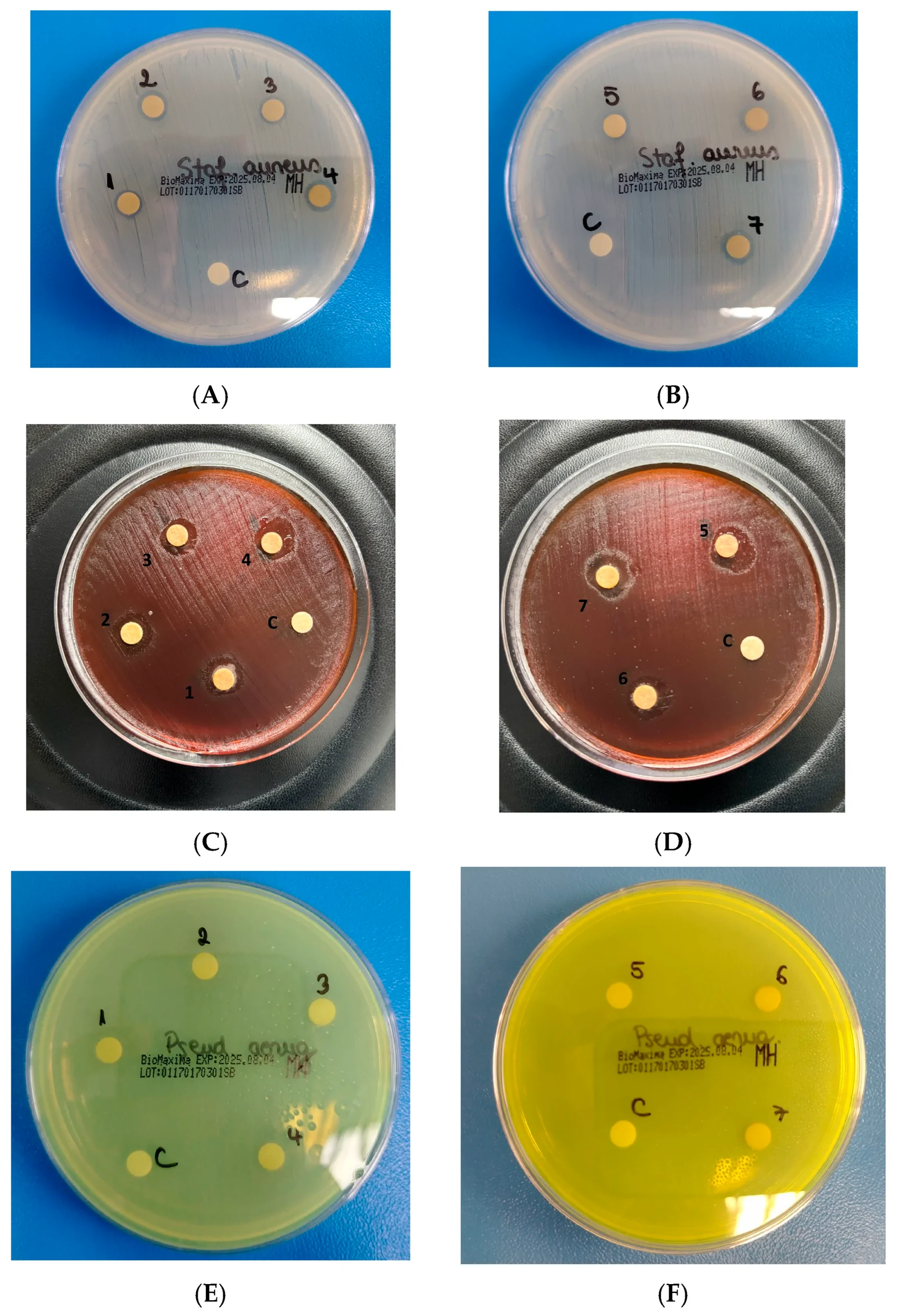
The antimicrobial activity of ACM 1–7. Growth inhibition zones of (**A**,**B**) *Staphyloccocus aureus*; (**C**,**D**) *Streptoccocus pneumoniae*; (**E**,**F**) *Pseudomonas aeruginosa*.

The antimicrobial evaluation of the mixtures revealed moderate inhibition against Gram-positive bacteria. The strongest effects were recorded for formulations containing higher proportions of M.O. or balanced ratios of the three species. Specifically, ACM4 and ACM5 produced inhibition zones of 20 mm against *Streptococcus pneumoniae*; ACM4, ACM5, and ACM6 exhibited the highest inhibitory effect against *Staphylococcus aureus* (10 mm). None of the mixtures showed inhibitory activity against *Pseudomonas aeruginosa*. In Table 6 are present the results and Figure 1 present the growth inhibition zones. 

## 3. Discussion

### 3.1. The Content of Polyphenols, Flavonoids and Anthocyanin in the Studied Plant Extracts

The extract derived from *Arnica montana* L. flowers exhibited the highest total polyphenol content; this fact suggests that A.M. may possess a stronger antioxidant potential, given that polyphenols are among the main compounds responsible for scavenging reactive oxygen species (ROS) and protecting cellular components from oxidative stress [9,10]. Regarding total flavonoids, A.M. again showed the highest concentration (24.53 mg QE/g DW), whereas M. officinalis and C.B.P. presented slightly lower values (20.08 and 18.84 mg QE/g DW, respectively). Flavonoids play an essential role in modulating anti-inflammatory and antimicrobial activities [11], and the elevated levels found in A.M. further indicate their pharmacological relevance.

Anthocyanins are known for their antioxidant and cytoprotective properties, contributing to the overall biological activity of plant extracts [12,13].

Overall, the results highlight *Arnica montana* L. as the richest source of phenolic compounds among the studied species, which supports its use as a promising natural antioxidant. Both *Capsella bursa-pastoris* Medik. and *Melissa officinalis* L. also demonstrated considerable levels of polyphenols and flavonoids, consistent with previous reports describing interspecific variation in secondary metabolite content [14,15].

### 3.2. Antioxidant Capacity for Capsella bursa-pastoris Medik., Arnica montana L., Melissa officinalis L. Species

In this study, the antioxidant capacity of *Capsella bursa-pastoris* Medik. (C.B.P), *Melissa officinalis* L. (M.O), and *Arnica montana* L. (A.M) was evaluated using four complementary in vitro assays (DPPH, CUPRAC, ABTS, and FRAP). These assays reflect different reaction mechanisms—radical scavenging and electron transfer—and together provide a comprehensive profile of antioxidant potential.

Among the extracts investigated, A.M. consistently demonstrated the strongest antioxidant performance, followed by M.O. and C.B.P. The markedly higher DPPH radical-scavenging activity observed for A.M. and M.O., compared with C.B.P., reflects their superior hydrogen-donating capacity. This trend was further supported by the CUPRAC and ABTS assays, in which A.M. and M.O. exhibited substantially greater Trolox-equivalent antioxidant capacities than C.B.P. Interestingly, in the FRAP assay, C.B.P. approached the ferric-reducing ability of A.M., suggesting the presence of specific constituents in C.B.P. that are particularly effective in electron-transfer-based redox reactions. These findings indicate that, while A.M. and M.O. display broad-spectrum radical-scavenging activity across multiple mechanisms, C.B.P. may contain specialized phenolic compounds that perform disproportionately well in assays involving ferric ion reduction.

The observed trends align with reports in the recent literature. M.O. extracts are consistently described as rich in phenolic acids (rosmarinic, caffeic) and flavonoids, conferring strong DPPH and ABTS scavenging capacity [16,17]. Adamczyk-Szabela et al. (2023) demonstrated that environmental stress increases both the phenolic content and antioxidant activity of M.O., confirming its robust redox potential [18]. Likewise, nano-ethosomal formulations of M.O. maintained high DPPH activity and strong metal-chelating ability [19]. *Arnica montana* L. is also recognized for its rich phenolic composition, including chlorogenic and caffeoylquinic acids, which are responsible for its potent antioxidant and anti-inflammatory properties [20]. Petrova et al. (2025) reported that elicitation with yeast extract or salicylic acid enhanced A.M.’s phenolic content and antioxidant capacity, consistent with the high values observed here [21]. Although *Capsella bursa-pastoris* Medik. has been less extensively studied, recent research confirms its significant antioxidant potential. A 2022 comparative analysis of Himalayan herbs found that CBP ethanolic extract exhibited moderate DPPH and ABTS activity relative to more polyphenol-rich species [22]. Other works attribute C.B.P.’s antioxidant power mainly to flavonoids (quercetin, kaempferol) and phenolic acids, which may account for its strong FRAP response in our study [23]. The slight differences among assays can be explained by the distinct redox chemistry each test probes—DPPH and ABTS reflecting radical-scavenging, while FRAP and CUPRAC indicate metal-reducing efficiency [24].

The strong performance of A.M. and M.O. likely results from higher total phenolic and flavonoid concentrations, which are directly correlated with antioxidant potential [25]. The relatively elevated FRAP value for C.B.P. suggests enrichment in specific reducing agents, possibly sulfur-containing or nitrogenous compounds typical of Brassicaceae plants [26]. Moreover, C.B.P. is known to contain glucosinolates and their hydrolysis products, which can participate in redox reactions and contribute to overall antioxidant behavior [27].

### 3.3. LC-MS Analysis

The results obtained by LC/MS analysis highlighted the presence and quantification of phenolic compounds and flavonoids with major biological relevance, recognized for their antioxidant, anti-inflammatory and antimicrobial potential. Both *Arnica montana* L. and *Melissa officinalis* L. demonstrated a complex phytochemical profile, but with notable quantitative differences.

*Arnica montana* L. extract was found to have a high content of luteolin-7-O-glucoside (121.8 µg/mL) and tilianin (11.7 mg/mL). Luteolin and its derivatives have been reported to have antibacterial activity against *Streptococcus mutans* and *Porphyromonas gingivalis*, agents involved in the etiology of caries and periodontal disease [28,29]. In addition, luteolin inhibits the production of IL-6 and TNF-α, cytokines involved in gingival inflammation [30]. Tilianin, a glycosidic flavonoid, is described for its antioxidant properties and protective effects on vascular tissues [31], suggesting a possible adjuvant role in reducing oxidative stress present in several conditions.

*Melissa officinalis* L. showed lower concentrations for most compounds but stood out with an increased content of acacetin (3.26 µg/mL). Compounds from the flavonoid family (including acacetin) have been documented to have antifungal activities against *Candida albicans* and anti-inflammatory effects [32,33]. M.O. is also a known source of phenolic acids (chlorogenic acid, caffeic acid), compounds that may contribute to inhibiting dental demineralization and the development of bacteria. Comparatively, a higher concentration of quercetin (22.8 µg/mL) was identified in A.M. than in M.O. (1.7 µg/mL). Quercetin has well-documented antimicrobial and anti-inflammatory properties, with potential for use in adjuvant treatments for periodontitis [34]. The presence of phenolic compounds—chlorogenic acid (4.02 µg/mL in A.M.; 0.95 µg/mL in M.O.) and caffeic acid (0.33 µg/mL in *Arnica montana* L.; 0.24 µg/mL in *Melissa officinalis* L.) complements biological activity through antioxidant effects and action against cariogenic bacteria [35].

It should be noted that certain substances, such as apigenin, luteolin-7-O-rutinoside and vitexin, were below the limit of quantification in *Melissa officinalis* L. or were not detected in *Arnica montana* L. However, the literature shows that apigenin, even at low concentrations, can inhibit biofilm formation and glucan production by *Streptococcus mutans* [36], so their absence in the extracts analyzed does not exclude relevant biological potential in other phytotherapeutic preparations, such as bacterial biofilm [37].

Overall, the results support the hypothesis that *Arnica montana* L. and *Melissa officinalis* L. extracts may represent valuable sources of bioactive compounds with a potential adjuvant role in the prevention and management of various diseases. However, the quantitative differences observed between species highlight the need for extract standardization and further in vitro and in vivo studies to confirm their biological relevance and potential clinical applicability.

LC–MS analysis of *Capsella bursa-pastoris* Medik. revealed a phytochemical profile dominated by phenolic acids, particularly chlorogenic acid and its isomers, followed by caffeic acid, which were present in substantial amounts. These major compounds are likely the primary contributors to the extract’s antioxidant activity, due to their well-established capacity to scavenge free radicals and act as reducing agents, as observed in the FRAP assay [5]. Their high abundance may explain the strong and stable reduction in reactive oxygen species compared to other plant extracts with more evenly distributed phytochemical profiles.

Although flavonoids such as quercetin, tilianin, and luteolin-7-O-glucoside were detected at lower concentrations, they may still exert a supportive antioxidant effect and modest antimicrobial activity through membrane interactions or enzyme inhibition [14]. However, their relatively low abundance likely accounts for the weaker antimicrobial activity of C.B.P. compared to extracts richer in flavonoids.

In summary, the biological effects of C.B.P. appear to be primarily driven by the major phenolic acids, with flavonoids playing a secondary, complementary role. This distribution suggests that the antioxidant potential and partial antimicrobial activity of the extract correlate directly with the compounds present in the highest concentrations, while minor constituents exert only a limited influence.

### 3.4. Antimicrobial Activity for Capsella bursa-pastoris Medik., Arnica montana L., Melissa officinalis L. Species

The results obtained were found to be consistent with previous reports on the antimicrobial properties of these plants. M.O. is rich in phenolic acids (rosmarinic acid, caffeic acid), flavonoids, and monoterpenes, such as citral and citronellal, which are known to disrupt microbial membranes and inhibit quorum sensors [37]. Several studies have reported strong antibacterial activity of M.O. extracts and a strong inhibition against Staphylococcus aureus [38]. Our results corroborate these observations, confirming that M.O. is particularly effective against Gram-positive bacteria.

A.M. exhibited moderate activity, consistent with previous studies reporting inhibition of Staphylococcus aureus and other oral pathogens by A.M. flower extracts [39]. The bioactivity of A.M. is attributed to sesquiterpene lactones (e.g., helenalin) and flavonoids, which possess antibacterial, antifungal, and antibiofilm properties [40]. The lower activity of C.B.P. relative to M.O. and A.M. aligns with studies showing that C.B.P extracts exert mainly anticandidal and antibiofilm effects at relatively high concentrations [41]. Nevertheless, the measurable inhibition observed in our study suggests that C.B.P. may still provide a valuable contribution in multi-herb formulations.

The inefficacy of all three extracts against *Pseudomonas aeruginosa* is unsurprising given the intrinsic resistance mechanisms of this Gram-negative pathogen, including efflux pumps and low outer-membrane permeability [42]. This highlights a common limitation of plant-derived antimicrobial activity, which is typically more effective against Gram-positive bacteria due to the absence of the outer-membrane barrier.

Following the determinations made on individual extracts, their mixtures were obtained and investigated in order to evaluate the impact of the combination on the phytochemical profile and biological activity.

### 3.5. Antioxidant Capacity for Mixture of Capsella bursa-pastoris Medik., Melissa officinalis L., Arnica montana L.

All tested mixtures exhibited considerable antioxidant activity, with inhibition values ranging between approximately 64% and 75%. Among them, the ACM4 mixture demonstrated the highest scavenging activity (75.34% at 0.1 mL extract), followed by ACM2 (72.68%) and ACM1 (71.48%). Increasing concentration beyond 0.3 mL did not significantly enhance the inhibitory effect, suggesting that the antioxidant compounds reached a saturation level at lower volumes.

These findings indicate that *Melissa officinalis* L., which is rich in rosmarinic acid and flavonoids, plays a major role in free radical scavenging, as reported by Oroian and Escriche [43] and Sipos et al. [44]. The observed trend is consistent with the polyphenolic content and synergistic effects among phenolic compounds from *Arnica montana* L. (notably sesquiterpene lactones) and *Capsella bursae-pastoris* L. (containing sinapic acid derivatives) [45,46].

Similar observations have been reported in polyherbal formulations, where synergistic ratios increased antioxidant efficacy up to 20% compared to single extracts [47,48]. These results suggest that combining A.M., C.B.P., and M.O. may lead to a formulation with both potent antioxidant properties and potential anti-inflammatory activity.

Although the antioxidant activity of the extracts was demonstrated using in vitro chemical assays, further studies involving cell-based models would be useful to better understand the biological relevance and mechanisms of action of the identified phenolic compounds.

### 3.6. Antimicrobial Activity for Mixture of Species Capsella bursa-pastoris Medik., Melissa officinalis L., Arnica montana L.

The present results demonstrate that the combinations of the three plant extracts (A.M., C.B.P., M.O.) diluted in 70% alcohol show moderate antimicrobial activity against Gram-positive bacteria, with varying efficacy depending on the ratio. The fact that ACM4 (1:1:2) and ACM5 (2:2:1) achieved the highest inhibition against *Streptococcus pneumoniae* suggests that increasing the proportion of one component relative to the others may enhance efficacy. One possible explanation is a synergistic effect between the extracts, in which a higher proportion of a more active extract either enhances penetration or disrupts microbial membranes more effectively.

Interestingly, in some cases, the 1:1 diluted extract produced smaller inhibition zones compared to certain diluted samples. This observation may be related to the reduced diffusion capacity of highly concentrated extracts. Increased viscosity and the presence of less diffusible compounds may limit radial diffusion, while moderate dilution may facilitate better diffusion of active antimicrobial constituents, resulting in larger inhibition zones. In addition, concentration-dependent physicochemical effects may also contribute to this behavior. At higher concentrations, partial aggregation or intermolecular interactions of extract constituents may reduce their effective bioavailability, even in the absence of visible precipitation. At moderate dilutions, improved dispersion of bioactive compounds may enhance their interaction with the microbial cells, leading to increased apparent activity.

This observation may also be explained by a hormetic dose–response relationship, in which maximal biological effects occur at intermediate rather than higher concentrations. In complex plant extracts, such behavior may reflect a shift from synergistic interactions at moderate concentrations to antagonistic effects or reduced bioavailability at higher doses. Similar non-linear responses have been reported in phytochemical studies and are consistent with the multifactorial nature of plant-derived mixtures.

These observations align with literature reporting that medicinal plant extracts often exhibit enhanced antimicrobial activity when used in combination due to multi-target mechanisms (e.g., cell wall disruption, efflux pump inhibition, biofilm interference) rather than classic antibiotic single-target action [49]. Moreover, the review by Oulahal & Degraeve (2022) highlights that mixtures of plant phenolic extracts may yield broader spectrum activity than single extracts alone [50].

The relatively lower activity against *Staphylococcus aureus* compared to *Streptococcus pneumoniae* suggests that *Streptococcus pneumoniae* may be more susceptible to these extract combinations, or that the extracts may preferentially affect targets more relevant in Streptococcus pneumoniae. These are known limitations of plant phenolic extracts: their in vitro antimicrobial activity may not translate directly to all microorganisms, especially Gram-negative bacteria, due to matrix effects or diffusion constraints [51].

In comparison to the antibiotic controls, the extract mixtures produced smaller inhibition zones than standard antibiotics; however, given the plant origin and crude nature of the extracts, these results are encouraging as preliminary screening. They suggest that extract combinations may serve as leads for further refinement, purification, and synergistic combination with antibiotics. Indeed, previous studies emphasize that plant-derived compounds may act synergistically with conventional antibiotics to overcome resistance.

The biological activity observed for the investigated extracts may be related to several classes of phytochemicals previously reported in *Arnica montana* L., *Capsella bursa-pastoris* Medik., and *Melissa officinalis* L. *Arnica montana* L. is known to contain sesquiterpene lactones (particularly helenalin and its derivatives), flavonoids such as quercetin and luteolin glycosides, and phenolic acids, which have been associated with anti-inflammatory, antimicrobial, and antioxidant activities [52]. *Capsella bursa-pastoris* Medik. contains a wide range of bioactive constituents, including flavonoids (quercetin, kaempferol derivatives), phenolic acids, glucosinolates, and biogenic amines, which have been reported to contribute to antioxidant and antimicrobial effects [53]. In *Melissa officinalis* L., the biological activity is commonly attributed to phenolic compounds such as rosmarinic acid, caffeic acid derivatives, and flavonoids, as well as volatile terpenoids including citral and geraniol, which are well documented for their antioxidant, antimicrobial, and anti-inflammatory properties [54,55]. However, it should be emphasized that the extracts used in this study represent complex phytochemical mixtures. Therefore, the observed bioactivity cannot be unequivocally attributed to a single compound or compound class. Instead, the effects may result from additive or synergistic interactions between multiple constituents present in the extracts. Since the present study did not compare the activity of the total extracts with isolated major compounds, attributing the observed biological effects exclusively to individual phytochemicals remains speculative and should be interpreted with caution. Further studies involving bioactivity-guided fractionation and testing of isolated constituents would be necessary to clarify the contribution of specific compounds.

## 4. Materials and Methods

### 4.1. Plant Material

The samples of *Capsella bursa-pastoris* Medik. (C.B.P.), *Melissa officinalis* L. (M.O.), used in this study were collected in 2023 from the spontaneous flora of Bihor counties, in unpolluted areas. C.B.P was collected between April and June, and M.O. in June, both from unpolluted areas of Oradea city (Bihor County, Romania). A.M. was purchased from Farmacia Parmelia, Hidisel, Biror County, Romania in November 2024. It is available as arnica flower tea, sold by Stefmar, Ramnicu Vâlcea, Romania and is valid until 2026.

The soil type from which C.B.P and M.O. were collected is classified as preluvosol, with a clayey texture and rich in carbonates. A specimen of each species was kept in the herbarium of the Faculty of Medicine and Pharmacy Oradea, Romania, registered in NYBG Steere Herbarium as follows:

*Capsella bursa-pastoris* Medik.—U0P 05.136.

*Melissa officinalis* L.—U0P 05.707.

For C.B.P., it has been demonstrated in another research article that the aerial part has the highest amount of active ingredients, so the aerial part of the plant was used to make the determinations and extracts [56]. Only the leaves were used for M.O., and only the flowers were used for A.M., because these parts of the plants are the richest in bioactive compounds [57].

For chemical analysis, the plant materials of *Capsella bursa-pastoris* Medik., *Arnica montana* L., and *Melissa officinalis* L. were each processed individually. The materials were dried at an average temperature of 40 °C for 96 h.

Extracts with a concentration of 10% (*w*/*w*) were then prepared for each plant by macerating the dried material in 70% (*v*/*v*) ethanol. The plant materials were allowed to macerate for 10 days in a cool, dark environment to preserve their bioactive compounds.

After the maceration period, each mixture was filtered, and the resulting solutions were stored at 6 °C ± 2 °C. Five days later, the extracts were carefully decanted, and various mixtures were subsequently prepared for further analysis.

After filtration, the solvent was partially removed using a Heidolph rotary evaporator Savski Nasip 7, Belgrade, Serbia in order to evaporate the ethanol and obtain concentrated crude extracts. The remaining aqueous residues were subsequently diluted with ethyl alcohol to obtain the desired concentrations for the phytochemical and biological assays.

The extract concentrations were defined based on the initial 10% (*w*/*w*) preparation, and serial dilutions (1:1, 1:2, 1:4, and 1:6) were performed. The resulting concentrations were calculated proportionally according to the dilution principle (C_1_V_1_ = C_2_V_2_) and expressed as relative concentrations, yielding final values of 5%, 3.33%, 2%, and 1.43%, respectively. This approach ensured a controlled and reproducible framework for evaluating dose-dependent effects. The dilutions are presented in Table 7.

Since all extracts exhibited the most effective antimicrobial activity at a 1:4 dilution, the mixtures were prepared using extracts diluted in a 1:4 ratio.

After analyzing each species individually, we observed that all extracts exhibited the most effective antimicrobial activity at a 1:4 dilution. The mixtures were prepared using extracts diluted in a 1:4 ratio (2%), as shown in Table 8 and ACM 1–7 are the abbreviations for the mixtures.

### 4.2. Phytochemical Analysis

Phytochemical analysis of plants is essential for identifying, quantifying, and characterizing secondary compounds (alkaloids, flavonoids, tannins, terpenoids, glycosides, etc.) that determine the biological and pharmacological properties of the species studied. The following tests were performed through phytochemical analysis: determination of total polyphenols, flavonoids, and anthocyanins contained in plants and the antioxidant capacity of individual plants and then of the plant mixture.

#### 4.2.1. Determination of Total Polyphenol Content

The total polyphenol content of the samples was determined using the Folin–Ciocalteu method. This colorimetric technique relies on the oxidation of phenolic hydroxyl groups in an alkaline environment, facilitated by sodium carbonate. The resulting blue coloration has an absorbance that increases proportionally with the number of phenolics OH groups and is measured at 765 nm.

**Figure 2:**
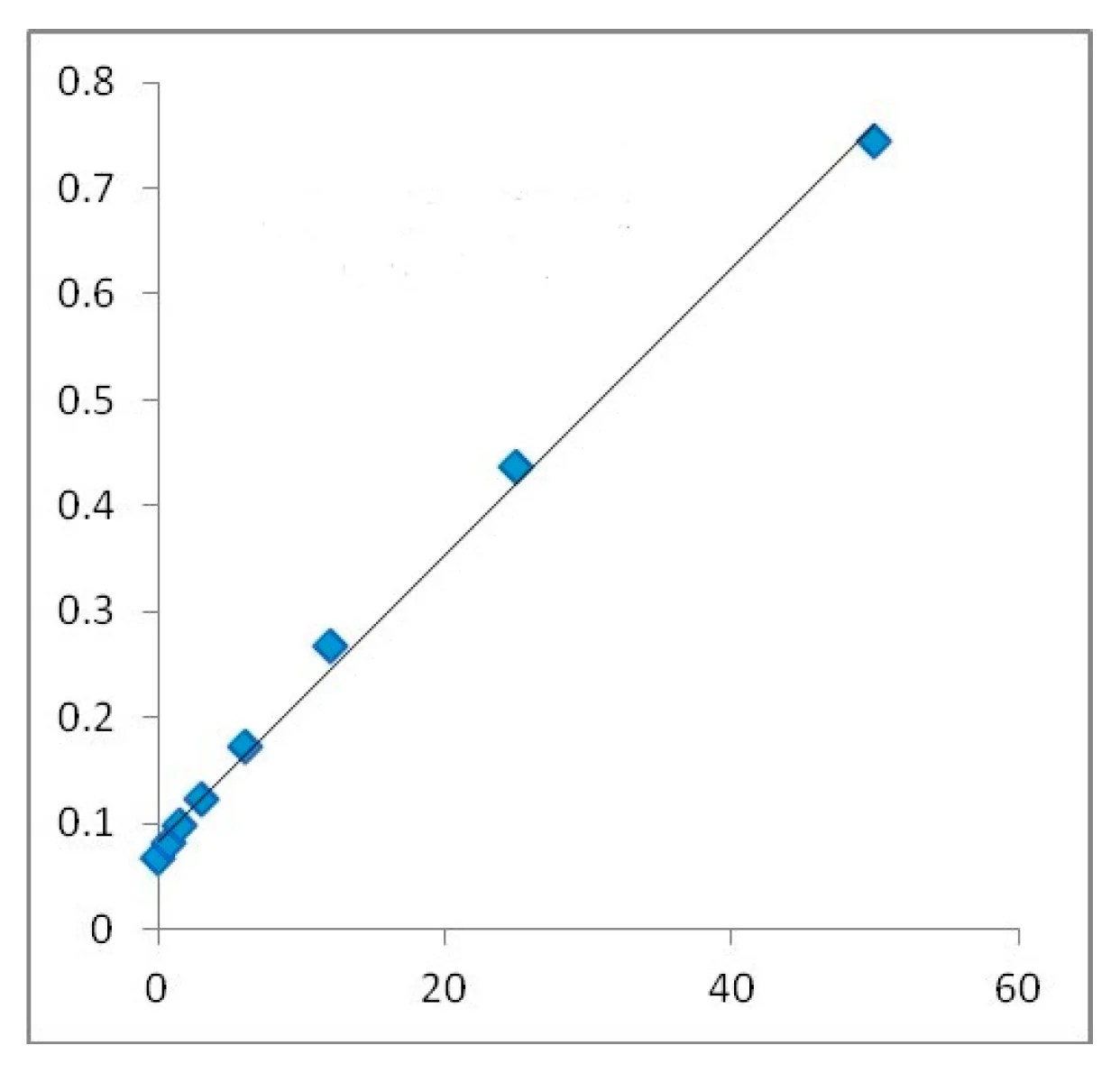
Calibration line made with gallic acid for Folin–Ciocalteu method in alcoholic medium. The absorbance was 765 nm (concentration of gallic acid mg/GAE/100 g DW), where the blue squares highlight the mg/mL values used for the regression equation.

For each measurement, 0.1 mL of extract solution (containing 1000 μg of the sample) was added to a 50 mL volumetric flask containing 46 mL of distilled water. Subsequently, 1 mL of Folin–Ciocalteu reagent (Merck) was introduced, and the flask was shaken thoroughly to ensure mixing. After a 3 min reaction period, 3 mL of a 2% sodium carbonate (Na_2_CO_3_) aqueous solution was added. The mixture was then incubated at room temperature for 2 h. Absorbance was measured at 765 nm using a Shimadzu UV-1700 PharmaSpec UV–VIS Spectrophotometer (UV-1700 PharmaSpec, Shimadzu, Kyoto, Japan). A calibration curve was constructed using standard gallic acid solutions prepared and analyzed under the same conditions [58,59]. The calibration line (Figure 2) was created using quercetin (QE) as its standard.

#### 4.2.2. Determination of Total Flavonoids

**Figure 3:**
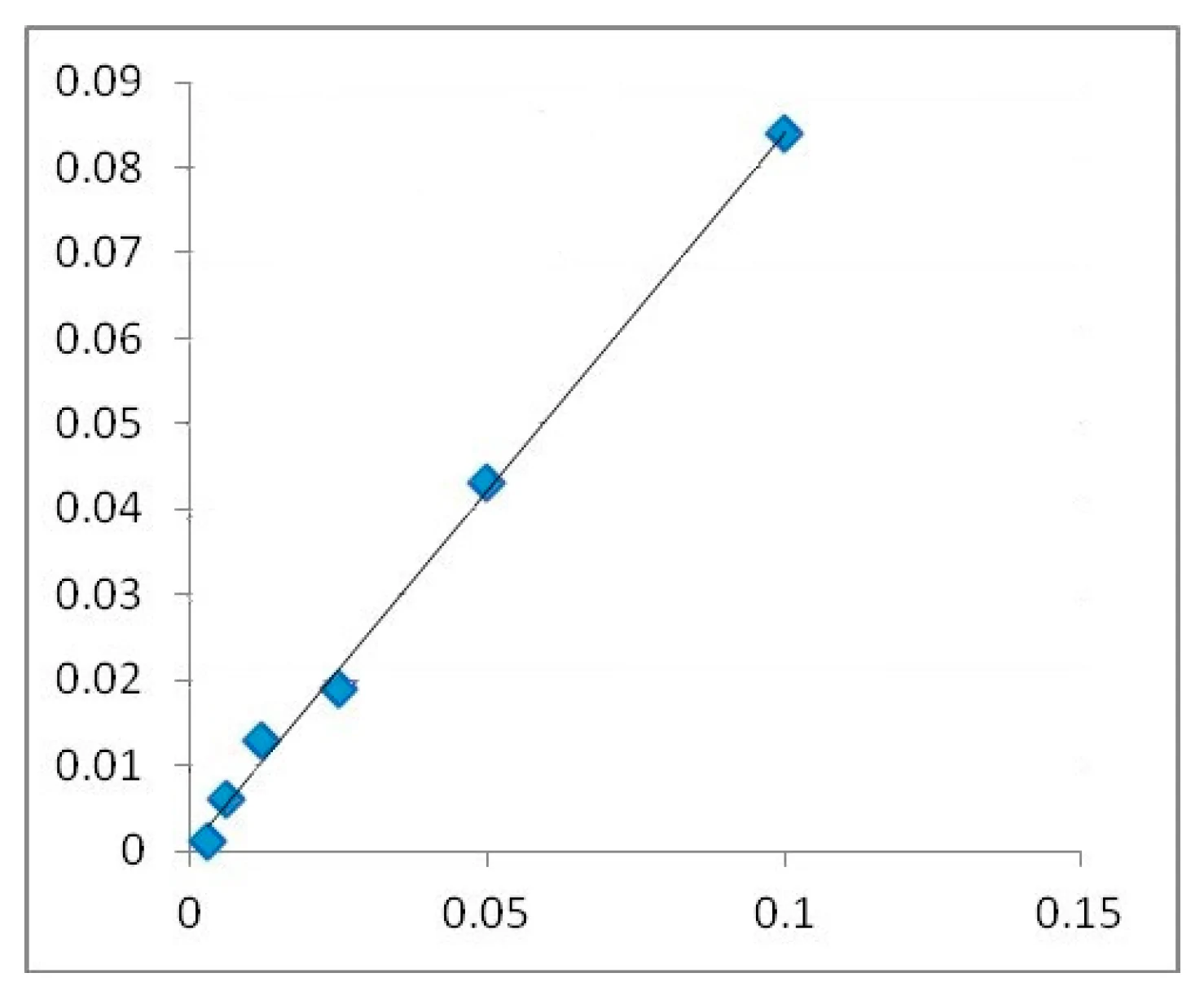
Calibration line made with quercetin in alcoholic medium (surroundings, environment). The absorbance was 510 nm (concentration of quercetin mg QE/g DW).), where the blue squares highlight the mg/mL values used for the regression equation.

Total flavonoid content was estimated using a modified colorimetric technique as previously reported [60]. To begin, 1 mL of the sample solution (0.1 mg/mL extract) was added to a 10 mL volumetric flask, followed by 4 mL of distilled water. Subsequently, 3 mL of a 5% sodium nitrite (NaNO_2_) solution was introduced. After a five-minute interval, 0.3 mL of a 10% aluminum chloride (AlCl_3_) solution was added. Six minutes later, 2 mL of 1 M sodium hydroxide (NaOH) was incorporated. The mixture was then brought up to volume with distilled water and mixed thoroughly. The absorbance of the final solution was measured at 510 nm using a Shimadzu UV-1700 Pharmaspec UV–Visible spectrophotometer [61,62,63]. This procedure was also applied to the standard solutions of quercitin, which resulted in a standard line (Figure 3).

#### 4.2.3. Determination of Total Anthocyanin Content

The total anthocyanin concentration was evaluated using a spectrophotometric approach adapted from the method outlined by Connor et al. (2002) [62]. This technique relies on the pH-dependent color variation in anthocyanin molecules, which allows for their quantification even in complex mixtures where other pigments or degradation products may be present. The method is based on the structural transformation of the anthocyanins in response to pH. At pH 1.0, anthocyanins primarily exist in their colored flavylium cation (oxonium) form, whereas at pH 4.5, they convert to a nearly colorless hemiketal (chalcone-like) structure. Measuring the difference in absorbance at these two pH levels provides an accurate estimation of total monomeric anthocyanin content.

The reagents were 0.025 M potassium chloride buffer, pH 1.0, 0.4 M sodium acetate buffer, pH 4.5, and methanol with 0.3% hydrochloric acid (*v*/*v*). Approximately 0.15 g of sample was weighed and homogenized in acidified methanol using an Ultraturrax homogenizer (IKA Works GmbH & Co. KG, Staufen im Breisgau, Germany) at 3000 rpm for 1 min. The homogenate was centrifuged at 5000 rpm for 20 min. The remaining solid was re-extracted twice as much under the same conditions, and all supernatants were combined for analysis. The pooled extract was diluted in two buffer systems: one at pH 1.0 (KCl buffer) and the other at pH 4.5 (acetate buffer), using a 5:95 (*v*/*v*) ratio. The dilutions were left to equilibrate at room temperature for 15 min. Absorbance was then recorded at the maximum visible wavelength (λ_vis-max, typically 530 nm) and at 700 nm to correct for haze or turbidity, using the respective buffers as blanks [64].

Corrected absorbance (A) was calculated using the following equation:

A = (A λvis-max − A700) pH 1.0 − (A λvis-max − A700) pH 4.5(1)

The concentration of monomeric anthocyanins (expressed as cyanidin-3-glucoside equivalents) was calculated with:

Monomeric anthocyanins (mg/L) = (A × MW × DF × 1000)/(ε × 1)(2)

where A is the absorbance calculated from Equation (1); MW is the molecular weight of cyanidin-3-glucoside (449.2 g/mol); DF is the dilution factor; ε is the molar absorptivity (26,900 L·mol^−1^·cm^−1^); 1 is the path length of the cuvette in cm.

Final results were expressed as milligrams of cyanidin-3-glucoside per 100 g of dry weight (mg C3G/100 g DW).

#### 4.2.4. Antioxidant Capacity

##### Ferric-Reducing Antioxidant Power (FRAP) Assay

The antioxidant capacity of the samples was evaluated using the Ferric-Reducing Antioxidant Power (FRAP) assay, as originally described by Benzie and Strain (1996) [65]. This method quantifies the ability of antioxidants in the sample to reduce the ferric-tripyridyltriazine complex (Fe^3+^-TPTZ) to its ferrous form (Fe^2+^-TPTZ) under acidic conditions, producing a blue-colored chromophore with maximum absorbance at 595 nm. Three stock solutions were prepared: 300 mM acetate buffer (pH 3.6); FeCl_3_·6H_2_O solution: 270 mg of ferric chloride hexahydrate dissolved in 50 mL of distilled water; and TPTZ solution: 150 mg of 2,4,6-tripyridyl-s-triazine dissolved in 150 µL of HCl and brought to volume with 50 mL of distilled water.

The FRAP working reagent was freshly prepared by mixing 50 mL of acetate buffer, 5 mL of the FeCl_3_·6H_2_O solution, and 5 mL of the TPTZ solution. This reagent was incubated at 37 °C before use. For quantification, Trolox (a vitamin E analogue) was used as a standard antioxidant. A standard curve was constructed using Trolox concentrations ranging from 0 to 300 µM, resulting in a linear calibration curve with a correlation coefficient (R^2^) of 0.9956 and a regression equation of:

y = 0.0017x + 0.0848, where y represents the absorbance measured at 595 nm [66].

Sample absorbance was determined at 595 nm after mixing with the FRAP reagent. Antioxidant capacity was expressed as micromoles of Trolox equivalents per 100 µL of extract (µmol TE/100 µL) [67].

##### Cupric Ion (Cu^2+^)-Reducing CUPRAC Assay

The cupric ion-reducing antioxidant capacity of the plant extracts was evaluated using a modified version of the method described by Karaman et al. (2010) [68]. In this procedure, 0.25 mL of CuCl_2_ solution (0.01 M), 0.25 mL of neocuproine solution in ethanol (7.5 × 10^−3^ M), and 0.25 mL of ammonium acetate buffer (1 M) were sequentially added to a test tube. This mixture was then combined with the plant extract. The total volume was brought up to 2 mL with distilled water, and the contents were thoroughly mixed. The reaction mixtures were kept at room temperature for 30 min in stoppered tubes. After incubation, the absorbance was recorded at 450 nm against a reagent blank. An increase in absorbance signifies a higher antioxidant capacity through greater cupric ion (Cu^2+^) reduction [68,69,70].

##### Free Radical-Scavenging Method (DPPH) According to Brand-Williams et al. (1995) [7]

The radical-scavenging capacity of plant extracts against the stable free radical 2,2-diphenyl-1-picrylhydrazyl (DPPH) was evaluated following a slightly modified procedure based on Brand-Williams et al. (1995) [7]. In this assay, antioxidants present in the extracts reduce DPPH radicals by donating hydrogen atoms, causing a color change from deep violet to pale yellow. This change was monitored spectrophotometrically at 517 nm using a UV–Vis spectrophotometer. A fresh DPPH solution (6.0 × 10^−5^ M in methanol) was prepared daily to ensure consistency. Reaction mixtures were incubated in the dark at room temperature for 15 min before absorbance measurements. All samples were analyzed in triplicate to ensure reproducibility. The percentage of radical-scavenging activity was calculated using the formula:

% Inhibition = [(AB − AA)/AB] × 100

where AB = absorption of blank sample (t = 0 min), and AA = absorption of test extract solution (t = 15 min) [22,23].

##### ABTS Method

The ABTS assay is based on the ability of antioxidants to quench the ABTS radical cation (ABTS•^+^; 2,2′-azinobis-(3-ethylbenzothiazoline-6-sulfonic acid)), a green–blue chromophore exhibiting maximum absorbance at 734 nm. The ABTS•^+^ radical was generated by reacting an ABTS solution (7 mM) with potassium persulfate (2.45 mM) at room temperature, followed by incubation in the dark for 16 h. The antioxidant capacity of the samples was evaluated in comparison with the Trolox standard. Briefly, 0.1 mL of the sample extract was mixed with 0.9 mL of the ABTS•^+^ solution, and the absorbance was measured at 734 nm after 30 min of incubation.

Results were expressed as µmol Trolox equivalents per mL of extract. The ABTS values were calculated using the calibration curve:

y = 629x + 98.94 (R^2^ = 0.998),

where y represents the absorbance and x corresponds to the Trolox equivalent concentration (µmol) [71,72].

#### 4.2.5. LC-MS Analysis

The LC-MS (liquid chromatography–mass spectrometry analysis) was conducted using a Shimadzu Nexera I LC/MS-8045 UHPLC system (Kyoto, Japan), equipped with a quaternary pump, autosampler, electrospray ionization (ESI) source, and a quadrupole rod mass spectrometer [73]. Chromatographic separation was achieved on a Luna C18 reversed-phase column (150 × 4.6 mm, 3 μm particle size, 100 Å pore size) from Phenomenex (Torrance, CA, USA) [74]. The column temperature was maintained at 40 °C throughout the analysis.

The mobile phase, outlined in Table 9 consisted of a gradient mixture of methanol (Merck, Darmstadt, Germany) and ultrapure water obtained using a Simplicity Ultra-Pure Water Purification System (Merck Millipore, Billerica, MA, USA) [75]. Formic acid (Merck, Darmstadt, Germany) of LC/MS grade was used as the organic modifier. The flow rate was set to 0.5 mL/min, and the total run time for each analysis was 36 min [76].

Detection was carried out using a quadrupole rod mass spectrometer equipped with an electrospray ionization (ESI) source, operated in both positive and negative multiple reaction monitoring (MRM) modes (see Standards Table, Table 9) [77]. The interface temperature was maintained at 300 °C. Nitrogen was employed as both the nebulizing gas (35 psi) and the drying gas (10 L/min). The capillary voltage was set to +3000 V.

Analytical standards (Table 9) were obtained from Phytolab (Vestenbergsgreuth, Germany). For each concentration level, 1 µL of standard solution was injected. Sample extracts were diluted 1:5 with methanol, and 1 µL of the diluted extract was injected in triplicate.

Identification of target compounds was based on comparison of the mass spectra and characteristic MRM transitions of the chromatographically separated peaks with those of the reference standards [78]. Quantification was performed using the primary transition for each analyte, and calibration curves were generated for this purpose. Table 9 presents the calibration equations, correlation coefficients (R^2^), and the calculated limits of detection (LOD) and quantification (LOQ).

### 4.3. Antimicrobial Activity

Antimicrobial activity of C.B.P, M.O., A.M. and mixtures of different ratios were evaluated using the disk diffusion method with standard methodology and by determining the minimum inhibitory concentrations (MICs).

The microorganisms used were as follows: *Staphylococcus aureus* (Gram-positive)—ATCC 25923, *Streptococcus pneumoniae*—ATCC 49619, and *Pseudomonas aeruginosa* (−)—ATCC 27853 (Gram-negative) [79].

Mueller–Hinton agar was used for *Staphylococcus aureus*, *Streptococcus pneumoniae* and *Pseudomonas aeruginosa*. Inocula were prepared by directly suspending colonies grown for 20 to 24 h in saline, adjusted to a 0.5 McFarland standard. Each strain was inoculated onto the appropriate culture medium using a sterile cotton swab, and the plates were dried for 10 to 15 min. Sterile microdiscs 6 mm in diameter saturated with a 1:20 diluted extract of C.B.P., M.O., A.M., mixtures of different ratios of plants, and mixtures of these plants in the proportions described previously were placed onto the inoculated plates. After overnight incubation at 35.9 °C, inhibition zone diameters were measured in millimeters. Microdiscs impregnated with ethanol 70% (20 μL) were used as negative controls. All antimicrobial assays were carried out in triplicate to ensure the reliability and reproducibility of the results. Each experiment was independently repeated three times using freshly prepared bacterial cultures. The diameters of the inhibition zones were measured and expressed as mean values ± standard deviation (SD) [80,81].

## 5. Conclusions

The ethanolic extracts of *Arnica montana* L., *Melissa officinalis* L. and *Capsella bursa-pastoris* Medik. demonstrated high levels of polyphenols and flavonoids with relevant antioxidant activity. Among them, *Arnica montana* L. showed the strongest antioxidant capacity and the richest phenolic profile. LC–MS analysis revealed characteristic compounds, including luteolin-7-O-glucoside and tilianin in *Arnica montana* L., acacetin in *Melissa officinalis* L., and rutin and hyperoside in *Capsella bursa-pastoris* Medik. Functional antioxidant assays and antimicrobial tests indicated synergistic effects, particularly in the ACM4 mixture.

These results support the potential development of pharmaceutical formulation.

## Figures and Tables

**Table 1:** The content of polyphenols, flavonoids and anthocyanin of the *Capsella bursa-pastoris* Medik (C.B.P.)., *Melissa officinalis* L (M.O.). and *Arnica montana* L. (A.M.)

Bioactive Compounds	C.B.P. Herba	M.O. Leaves	A.M. Flowers
Content in total polyphenols (mg GAE */g DW)	368.17	352.73	426.12
Total flavonoids (mg QE **/g DW)	18.84	20.08	24.53
Anthocyanin (mg cyanidin/100 g DW)	2.7013	1.1250	3.4740

* GAE: gallic acid; ** QE: quercetin.

**Table 2:** Antioxidant activity determined by the four chemical methods of the *Capsella bursae pastoris* Medik., *Melissa officinalis* L. and *Arnica montana* L.

Part of the Plant	DPPH %	CUPRAC (μmol Trolox Equivalent/mL)	ABTS (μmol Trolox Equivalent/mL)	FRAP (μmol Trolox Equivalent/100 g)
C.B.P.	84	358	280	617
M.O.	89	516	314	573
A.M.	92	538	331	632

**Table 3:** LC-MS analysis for *Arnica montana* L., *Melissa officinalis* L. and *Capsella bursae pastoris* Medik.

Name of Identified and Quantified Compound	UM	A.M.	M.O.	C.B.P.
Caffeic acid	μg/mL	0.333 ± 0.0005	0.238 ± 0.0139	2.324 ± 0.0123
Chlorogenic acid	μg/mL	4.026 ± 0.0090	0.946 ± 0.0171	4.015 ± 0.0075
Crypto-Chlorogenic acid	μg/mL	2.509 ± 0.0056	0.830 ± 0.0094	1.081 ± 0.1260
Neo-Chlorogenic acid	μg/mL	0.923 ± 0.0105	0.145 ± 0.0100	1.097 ± 0.0198
Acacetin	μg/mL	2.059 ± 0.0470	3.259 ± 0.0585	0.310 ± 0.1032
Apigenina	μg/mL	<QL	<QL	5.173 ± 0.0534
Hesperetin	μg/mL	1.741 ± 0.0782	<QL	<QL
Luteolin-7-O-glucosid	μg/mL	121.800 ± 1.2329	11.495 ± 0.2888	7.305 ± 0.0550
Luteolin-7-O-rutosid	μg/mL	<QL	<QL	1.0471 ± 0.0071
Naringenin	μg/mL	0.564 ± 0.0090	<QL	<QL
Quercetin	μg/mL	22.807 ± 0.5472	1.698 ± 0.1232	12.445 ± 0.0520
Tilianin	μg/mL	11.707 ± 0.1649	1.161 ± 0.0472	6.357 ± 0.0065
Vitexin	μg/mL	Not identified	<QL	Not identified

**Table 4:** Antimicrobial activity of the tested samples (C.B.P., A.M. and M.O.) expressed as inhibition zone diameters (mm). Data are presented as mean ± standard deviation (SD) obtained from three independent experiments (*n* = 3). The solvent used for extract preparation was tested as negative control. No inhibition zone was observed.

Samples	UM/DISC (µL)	*Staphyloccocus aureus* Inhibition Zone (mm)	*Streptococcus pneumoniae* Inhibition Zone (mm)
M.O.:Ethanol 1:1 (5%)	20 µL	3 ± 0.1	Not detected
M.O.:Ethanol 1:2 (3.33%)	20 µL	5 ± 0.1	8 ± 0.2
M.O.:Ethanol 1:4 (2%)	20 µL	12 ± 0.3	15 ± 0.3
M.O.:Ethanol 1:6 (1.43%)	20 µL	10 ± 0.2	13 ± 0.3
A.M.:Ethanol 1:1 (5%)	20 µL	7 ± 0.2	3 ± 0.1
A.M.:Ethanol 1:2 (3.33%)	20 µL	15 ± 0.4	8 ± 0.2
A.M.:Ethanol 1:4 (2%)	20 µL	20 ± 0.4	18 ± 0.4
A.M.:Ethanol 1:6 (1.43%)	20 µL	10 ± 0.2	15 ± 0.3
CBP:Ethanol 1:1 (5%)	20 µL	2 ± 0.07	5 ± 0.1
CBP:Ethanol 1:2 (3.33%)	20 µL	6 ± 0.1	Not detected
CBP:Ethanol 1:4 (2%)	20 µL	12 ± 0.3	10 ± 0.3
CBP:Ethanol 1:6 (1.43%)	20 µL	10 ± 0.3	8 ± 0.2
Amoxicillin–clavulanic acid	2 µg	22	Not tested
Ampicilin	2 µg	18	28
Benzylpeniciline	1 µg	15	19
Ciprofloxacin	5 µg	24	25
Erytromycin	15 µg	26	29
Gentamicin	10 µg	22	Not tested
Levofloxacin	5 µg	26	24
Norfloxacin	10 µg	21	21
Oxacilin	1 µg	22	11
Ttraciclyne	30 µg	27	31

**Table 5:** Antioxidant activity determined by the four chemical methods involving the mixture in different ratios of *Capsella bursa-pastoris* Medik., *Melissa officinalis* L. and *Arnica montana* L.

Samples	Ratio A.M.:C.B.P.:M.O.	DPPH %	CUPRAC (μmol Trolox Equivalent/mL)	ABTS (μmol Trolox Equivalent/mL)	FRAP (μmol Trolox Equivalent/100 g)
ACM1	1:1:1	85	375	280	617
ACM2	2:1:1	87	528	314	573
ACM3	1:2:1	84	512	328	514
ACM4	1:1:2	88	542	335	632
ACM5	2:2:1	89	539	351	661
ACM6	2:1:2	87	494	317	574
ACM7	1:2:2	86	438	310	568

**Table 6:** Antimicrobial activity of the mixture extracts expressed as inhibition zone diameters (mm). Data are presented as mean ± standard deviation (SD) obtained from three independent experiments (*n* = 3). The solvent used for extract preparation was tested as negative control. No inhibition zone was observed.

Sample	Ratio AM:CBP:MO	UM/DISC (µL)	*Staphylococcus aureus* Inhibition Zone (mm)	*Streptococcus pneumoniae* Inhibition Zone (mm)	*Pseudomona aeruginosa*
ACM1	1:1:1	20 µL	8 ± 0.2	13 ± 0.4	Not detected
ACM2	2:1:1	20 µL	9 ± 0.2	16 ± 0.4	Not detected
ACM3	1:2:1	20 µL	8 ± 0.2	15 ± 0.4	Not detected
ACM4	1:1:2	20 µL	10 ± 0.3	20 ± 0.3	Not detected
ACM5	2:2:1	20 µL	10 ± 0.3	20 ± 0.4	Not detected
ACM6	2:1:2	20 µL	10 ± 0.2	16 ± 0.3	Not detected
ACM7	1:2:2	20 µL	9 ± 0.1	14 ± 0.2	Not detected
Amoxicillin–clavulanic acid	-	2 µg	22	Not tested	Not tested
Ampicilin	-	2 µg	18	28	Not tested
Benzylpeniciline	-	1 µg	15	19	Not tested
Ciprofloxacin	-	5 µg	24	25	29
Erytromycin	-	15 µg	26	29	Not tested
Gentamicin	-	10 µg	22	Not tested	20
Levofloxacin	-	5 µg	26	24	22
Norfloxacin	-	10 µg	21	21	Not tested
Oxacilin	-	1 µg	22	11	Not tested
Ttraciclyne	-	30 µg	27	31	Not tested

**Table 7:** Dilution for *Capsella bursa- pastoris* Medik., *Melissa officinalis* L. and *Arnica montana* L. with ethanol in different ratio and percentage concentrations.

Plant:Ethanol		Ratio (%)		
C.B.P.:Ethanol	1:1 (5%)	1:2 (3.33%)	1:4 (2%)	1:6 (1.43%)
M.O.:Ethanol	1:1 (5%)	1:2 (3.33%)	1:4 (2%)	1:6 (1.43%)
A.M.:Ethanol	1:1 (5%)	1:2 (3.33%)	1:4 (2%)	1:6 (1.43%)

**Table 8:** Presentation of the mixing ratio of 1:4 dilutions of the extracts: *Arnica montana* L. (A.M.), *Capsella bursa-pastoris* Medik. (C.B.P.) and *Melissa officinalis* L. (M.O.).

Mixtures	A.M.	C.B.P.	M.O.
ACM1	1	1	1
ACM2	2	1	1
ACM3	1	2	1
ACM4	1	1	2
ACM5	2	2	1
ACM6	2	1	2
ACM7	1	2	2

**Table 9:** Calibration curve equations, their correlation factors, and the determined limits of detection and quantification.

Name of Standard	Concentration Range, mg/mL	Calibration Curve Equation	Correlation Factor	Detection Limit, μg/mL	Quantification Limit, μg/mL
Caffeic acid	0.113–1.13	Area = 4.56317 ∗ 10^7^ ∗ conc[μg/mL] + 507,513	0.9908	0.02	0.04
Chlorogenic acid	0.14–1.40	Area = 4.53128 ∗ 10^8^ ∗ conc[μg/mL] − 2.63229 ∗ 10^6^	0.9753	0.02	0.03
Crypto-Chlorogenic acid	0.10–1.00	Area = 9.79604 ∗ 10^7^ ∗ conc[μg/mL] + 418,649	0.9989	0.01	0.02
Neo-Chlorogenic acid	0.10–1.00	Area = 2.08756 ∗ 10^8^ ∗ conc[μg/mL] + 81,773.3	0.9988	0.001	0.002
Acacetin	1.20–12.00	Area = 2.02177 ∗ 10^6^ ∗ conc[μg/mL] – 535,751	0.9532	1.06	1.59
Apigenin	0.105–1.05	Area = 7.72301 ∗ 10^6^ ∗ conc[μg/mL] + 500,152	0.9852	0.13	0.26
Hesperetin	1.00–10.00	Area = 828328 ∗ conc[μg/mL] + 249,156	0.9999	0.60	1.20
Luteolin-7-O-glucoside	0.285–2.85	Area = 2.50924 ∗ 10^6^ ∗ conc[μg/mL] + 376,997	0.9854	0.30	0.60
Luteolin-7-O-rutoside	5.70–57.00	Area = 1.29002 ∗ 10^6^ ∗ conc[μg/mL] + 6.69912 ∗ 10^6^	0.9886	10.39	20.77
Naringenin	0.16–1.60	Area = 2.87930 ∗ 10^6^ ∗ conc[μg/mL] + 187,922	0.9889	0.13	0.26
Quercetin	0.91–9.10	Area = 1.45460 ∗ 10^6^ ∗ conc[μg/mL] + 2.03435 ∗ 10^6^	0.9790	2.80	5.59
Tilianin	3.10–31.00	Area = 5880.86 ∗ conc[μg/mL] + 28,495.1	0.9901	9.69	19.38
Vitexine	0.10–1.00	Area = 4.38714 ∗ 10^6^ ∗ conc[μg/mL] + 309,385	0.9827	0.14	0.28

## Data Availability

The original contributions presented in the study are included in the article. Further inquiries can be directed to the corresponding author.
